# The Role of Muscle Biopsy in Diagnostic Process of Infant Hypotonia: From Clinical Classification to the Genetic Outcome

**DOI:** 10.3389/fneur.2021.735488

**Published:** 2021-10-05

**Authors:** Marco Veneruso, Chiara Fiorillo, Paolo Broda, Serena Baratto, Monica Traverso, Alice Donati, Salvatore Savasta, Raffaele Falsaperla, Maria Margherita Mancardi, Marina Pedemonte, Chiara Panicucci, Gianluca Piatelli, Mattia Pacetti, Andrea Moscatelli, Luca Antonio Ramenghi, Lino Nobili, Carlo Minetti, Claudio Bruno

**Affiliations:** ^1^Department of Neurosciences, Rehabilitation, Ophthalmology, Genetics, Maternal and Child Health, University of Genoa, Genoa, Italy; ^2^Paediatric Neurology and Neuromuscular Disorders Unit, Istituto di Ricovero e Cura a Carattere Scientifico Istituto Giannina Gaslini, Genoa, Italy; ^3^Center of Translational and Experimental Myology, Istituto di Ricovero e Cura a Carattere Scientifico Istituto Giannina Gaslini, Genoa, Italy; ^4^Metabolic and Neuromuscular Unit, A. Meyer Children's Hospital, Florence, Italy; ^5^Pediatric Clinic, Istituto di Ricovero e Cura a Carattere Scientifico Policlinico San Matteo Foundation, University of Pavia, Pavia, Italy; ^6^Neonatal Intensive Care Unit, Azienda Ospedaliera Universitaria San Marco-Policlinico, University of Catania, Catania, Italy; ^7^Child Neuropsychiatry Unit, Istituto di Ricovero e Cura a Carattere Scientifico Istituto Giannina Gaslini, Genoa, Italy; ^8^Unit of Neurosurgery, Istituto di Ricovero e Cura a Carattere Scientifico Istituto Giannina Gaslini, Genoa, Italy; ^9^Neonatal and Pediatric Intensive Care Unit, Istituto di Ricovero e Cura a Carattere Scientifico Istituto Giannina Gaslini, Genoa, Italy; ^10^Neonatal Intensive Care Unit, Istituto di Ricovero e Cura a Carattere Scientifico Istituto Giannina Gaslini, Genoa, Italy

**Keywords:** muscle biopsy, floppy infant, muscular dystrophy, congenital myopathy, genetic outcome

## Abstract

The role of muscle biopsy in the diagnostic workup of floppy infants is controversial. Muscle sampling is invasive, and often, results are not specific. The rapid expansion of genetic approach has made the muscle histopathology analysis less crucial. This study aims to assess the role and efficacy of muscle histopathology in the diagnostic algorithm of hypotonia in early infancy through a retrospective analysis of 197 infants who underwent muscle biopsy in their first 18 months of life. Data analysis revealed that 92/197 (46.7%) of muscle biopsies were non-specific (80) or normal (12), not allowing a specific diagnosis. In 41/197 (20.8%) cases, biopsy suggested a metabolic or mitochondrial myopathy, while in 23/197 cases (11.7%), we found evidence of muscular dystrophy. In 19/197 cases (9.7%), histopathology characteristics of a congenital myopathy were reported. In 22/197 cases (11.7%), the histopathological study indicated presence of a neurogenic damage. Overall, 46 diagnoses were then achieved by oriented genetic tests. Muscle biopsy results were consistent with genetic results in 90% of cases. Diagnostic algorithms for the diagnosis of a floppy infant are largely missing. Muscle biopsy alone can lead to a diagnosis, help the clinician in the choice of a genetic test, or even modify a diagnosis made previously.

## Introduction

Hypotonia in the neonatal period can be related to a central or peripheral condition (1). Approximately 80% of “floppy infants” have a primary central nervous system etiology (2, 3) such as genetic/chromosomal syndromes [Prader–Willi syndrome (4) or Down syndrome] or hypoxic–ischemic encephalopathy.

Peripheral motor unit disorders (myopathies, congenital myotonic dystrophy, and metabolic disorders) comprise another major diagnostic category (2, 3). Central and peripheral nervous system involvements are not mutually exclusive.

The diagnostic workup of infantile central hypotonia includes a broad spectrum of investigations, depending on the extent of the disease (5). Conversely, in the case of peripheral hypotonia, the battery of tests available to the clinician can promptly identify the anatomy of the pathologic processes in a more targeted way, for instance, a genetic test to exclude or confirm spinal muscular atrophy (SMA) or congenital myotonic dystrophy, serum creatine kinase (CK) dosage to point toward muscular dystrophy (6), and electrophysiological studies, which might show abnormalities in the nerves and, muscles, and disorders of the neuromuscular junction. Except for a few myopathies, normal electromyographic (EMG) findings suggest a central origin of the hypotonia (7).

The role of muscle biopsy in the diagnostic workup of floppy infants is controversial. The histopathology assessment is crucial for differentiating myopathies and muscular dystrophies. Muscle sample is, however, invasive; and often, results are not specific (8).

In addition, the rapid expansion in the knowledge of genetic disorders and availability of DNA-based diagnostic tests has made non-invasive and rapid diagnosis possible for several disorders, without needing a biopsy sample. As a matter of fact, specific DNA testing can be performed for myotonic dystrophy and SMA, which are the commonest cause of peripheral hypotonia of infants (9, 10).

The aim of this study is to assess the role and the efficacy of muscle histopathology in the diagnostic algorithm of hypotonia in early infancy through a retrospective analysis of 197 infants who underwent muscular biopsy in their first months of life.

## Methods

### Samples

Data relating to 197 muscle biopsy analyses from infants under 18 months of age with a diagnosis of floppy infant, performed at the Muscle Pathology Laboratory of the Gaslini Institute between February 2000 and November 2017, were collected.

Biopsies of 113 males and 84 females were studied. The average age at which the biopsies were performed is 6 months.

### Muscle Biopsy

All patients underwent open-air surgical sampling of the quadriceps muscle. All muscle biopsies were performed in the operating room under general anesthesia.

Fresh tissue muscle specimens were delivered to the Muscle Pathology Laboratory within 30 min of excision for freeze fixation using isopentane and liquid nitrogen.

Histological and histochemical methods were performed according to standard procedure in all cases but one in which no muscle tissue was available due to severe fatty replacement.

These included hematoxylin and eosin, Gomori's trichrome, myofibrillar ATPase (at pH 9.4, 4.6, and 4.3), cytochrome *c* oxidase (COX), succinate dehydrogenase (SDH), NADH-TR, periodic acid–Schiff (PAS), Oil Red O, acid phosphatase, esterase, and phosphorylase.

Immunohistochemical study was performed by indirect immunofluorescence on 54 samples, in particular in all biopsies, indicating muscular dystrophy (23) and in further 31 biopsy with unspecific findings. The following antibodies were tested: dystrophin COOH, dystrophin Mid Rod, dystrophin NH, alpha-sarcoglycan, beta-sarcoglycan, gamma-sarcoglycan, delta-sarcoglycan, alpha-dystroglycan, merosin, dysferlin, caveolin, and collagen 6.

A biochemical assay of the respiratory chain enzymes activity was performed according to Spinazzi et al. (11) on 31 biopsy samples either with specific findings for a mitochondrial defect or with non-specific characteristics.

Ultrastructural investigation was performed on three biopsy samples, all indicating a congenital myopathy.

### Statistical Analysis

For analysis of results, we inferred the biopsy query as indicated by the referring clinician and the conclusion as stated in the biopsy report. The biopsy reports were reviewed by two expert myopathologists (CF and CM). In all cases, the indication to perform the muscle biopsy was the presence of hypotonia.

On the whole study population, we calculated the relative prevalence of the different diagnostic categories as indicated by the conclusions of the histological report. Also, results from ancillary procedure were not considered for this classification.

According to main histopathology findings, we distinguished six different diagnostic categories:

- normal for age or not relevant changes.
- presence non-specific alterations (mild variability of fiber size and morphology, increased connective tissue, and presence of internal nuclei).
- metabolic and mitochondrial pathology (lipid or glycogen accumulation, ragged red fibers, COX defect, and enzymatic defect).
- congenital myopathy (when available classified in nemaline myopathy, myotubular or centronuclear myopathy, core myopathy, fiber-type disproportion, or congenital myopathy not further classified (i.e., predominance of type 1 fiber and fiber hypotrophy).
- muscular dystrophy (presence of necrosis, fibrotic substitution, marked variability of fiber size with hypo-atrophic fibers, hypertrophic fibers, splitting, and increased number of internal nuclei).
- neurogenic muscle damage (presence of grouped atrophy and type grouping).

Then, we calculated the prevalence rate of each category.

## Results

### Prevalence Analysis

**Figure 1:**
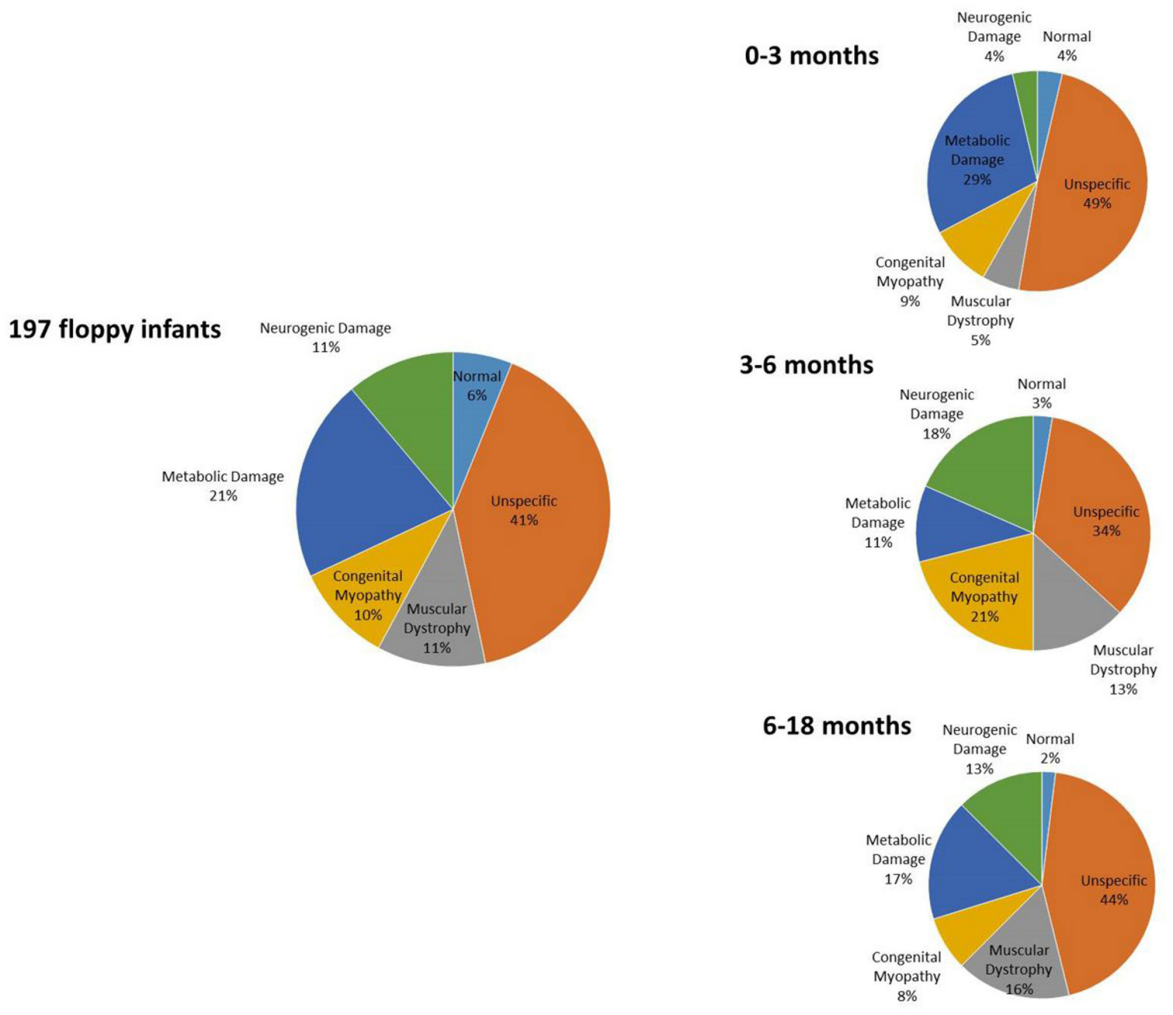
Distribution of histopathology diagnoses.

Results of prevalence analysis are summarized in Figure 1. Data analysis revealed that in 92/197 cases (46.7%), the study reported non-specific (80) or normal (12) biopsies, not allowing a specific diagnosis.

In 41/197 (20.8%) cases, biopsy suggested a condition of metabolic or mitochondrial pathology, specifically affecting oxidative metabolism (26), while in the remaining samples, we observed glycogen or lipid accumulation consistent with glycogen storage disorders (12) or lipid storage disorders (3), respectively.

In 23/197 cases (11.7%), we found evidence of congenital muscular dystrophy such as necrosis and fibrosis. Performing immunohistochemical analysis on these samples, we observed a condition of alpha-dystroglycan deficiency in six cases, a condition of merosin deficiency in six cases, and a condition of collagen VI deficiency in two cases.

In 19/197 cases (9.7%), the study reported histopathology characteristic of a congenital myopathy including one nemaline, one core, seven central nuclear, and five congenital fiber-type disproportion (CFTD). In five cases, unspecific findings such as predominance of type 1 fibers or fiber size variability were reported, thus not allowing the subclassification in a specific congenital myopathy.

Lastly, in 22/197 cases (11.7%), the histopathological study indicated presence of a neurogenic damage such as fiber-type grouping or grouped atrophy, suggesting a motor neuron disease.

A list of specific diagnoses is provided in Table 1.

**Table 1:** List of the different biopsy categories, including further specific findings at supplemental methods, age at biopsy, genetic tests, and result (when available).

**Sex**	**Age at biopsy (months)**	**Biopsy result**	**Notes**	**Genetic test**	**Genetic diagnosis**
F	6.3	Congenital muscular dystrophy	A-DG deficiency	Gene panel	*POMGNT1*
F	10	Congenital muscular dystrophy	Unspecific		
M	0.8	Congenital muscular dystrophy	Unspecific		
F	7	Congenital muscular dystrophy	Merosin deficiency	Single gene	*LAMA2*
F	10.4	Congenital muscular dystrophy	Unspecific		
M	4.4	Congenital muscular dystrophy	Merosin deficiency	Single gene	*LAMA2*
F	6.9	Congenital muscular dystrophy	Collagen VI deficiency	Single gene	*COL6*
M	7.6	Congenital muscular dystrophy	Merosin deficiency	Gene panel	No positive results
F	13.1	Congenital muscular dystrophy	Unspecific	WES	*TTN*
M	5.9	Congenital muscular dystrophy	A-DG deficiency	Gene panel	*GMPPB*
F	2.8	Congenital muscular dystrophy	Unspecific		
M	13.5	Congenital muscular dystrophy	A-DG deficiency	Single gene	No positive results
F	2	Congenital muscular dystrophy	Unspecific	Single gene	No positive results
nd	10.8	Congenital muscular dystrophy	Unspecific		
M	6.5	Congenital muscular dystrophy	Merosin deficiency; A-DG deficiency	Single gene	*LAMA2*
M	5	Congenital muscular dystrophy	Merosin deficiency	Single gene	No positive results
M	3.2	Congenital muscular dystrophy	Merosin deficiency		
M	7.3	Congenital muscular dystrophy	Collagen VI deficiency	Gene panel	No positive results
M	8.1	Congenital muscular dystrophy	A-DG deficiency	Gene panel	No positive results
M	15.6	Congenital muscular dystrophy	A-DG deficiency	Gene panel	*POMT2*
F	11.2	Congenital muscular dystrophy	A-DG deficiency	Gene panel	*POMGNT2*
M	2.1	Congenital myopathy	Centronuclear myopathy	Single gene	*MTM1*
M	0.6	Congenital myopathy	Congenital fiber-type disproportion		
F	5.3	Congenital myopathy	Unspecific		
F	7.7	Congenital myopathy	Unspecific	Gene panel	No positive results
M	3.7	Congenital myopathy	Centronuclear myopathy	Single gene	*MTM1*
M	3.5	Congenital myopathy	Central core disease	Gene panel	*RYR1*
M	8.5	Congenital myopathy	Nemaline myopathy	Single gene	*ACTA*
F	9.8	Congenital myopathy	Unspecific	Gene panel	*TNNT1*
M	0.4	Congenital myopathy	Centronuclear myopathy	Single gene	*MTM1*
M	8.7	Congenital myopathy	Congenital fiber-type disproportion	Single gene	No positive results
M	3.9	Congenital myopathy	Congenital fiber-type disproportion	Single gene	No positive results
F	4.2	Congenital myopathy	Unspecific	WES	*TPM3*
M	8.2	Congenital myopathy	Centronuclear myopathy	WES	*TTN*
M	4.3	Congenital myopathy	Centronuclear myopathy		
M	0.6	Congenital myopathy	Centronuclear myopathy	Single gene	No positive results
M	3.7	Congenital myopathy	Congenital fiber-type disproportion	Gene panel	
M	8.9	Congenital myopathy	Congenital fiber-type disproportion	WES	No positive results
F	1.6	Congenital myopathy	Unspecific	Gene panel	*CFL2*
F	13.8	Congenital myopathy	Centronuclear myopathy	Gene panel	*DNM*
M	11.3	Duchenne muscular dystrophy		Single gene	*DMD*
F	6.7	Limb-girdle muscular dystrophy	Sarcoglycanopathy	Gene panel	*SCGA*
M	6.4	Metabolic myopathy	Glycogen storage disease		
F	0.4	Metabolic myopathy	Glycogen storage disease		
M	8.6	Metabolic myopathy	Mitochondrial disease	Single gene	*AGK*
F	1.9	Metabolic myopathy	Glycogen storage disease		
F	12.9	Metabolic myopathy	Mitochondrial disease	Single gene	*POLG*
M	12.8	Metabolic myopathy	Mitochondrial disease		
F	3	Metabolic myopathy	Mitochondrial disease	Gene panel	*TSEN54*
M	4.1	Metabolic myopathy	Glycogen storage disease	Single gene	*PYGM*
M	13	Metabolic myopathy	Mitochondrial disease	MtDNA	
F	6.9	Metabolic myopathy	Mitochondrial disease	Single gene	*AGK*
M	7.6	Metabolic myopathy	Lipid storage disease		
F	7.5	Metabolic myopathy	Mitochondrial disease		
F	1.2	Metabolic myopathy	Glycogen storage disease		
F	0.4	Metabolic myopathy	Glycogen storage disease		
F	6.3	Metabolic myopathy	Lipid storage disease	MtDNA	No positive results
M	0.4	Metabolic myopathy	Glycogen storage disease	Single gene	No positive results
F	9	Metabolic myopathy	Glycogen storage disease		
M	9.4	Metabolic myopathy	Mitochondrial disease	MtDNA	No positive results
M	1.7	Metabolic myopathy	Glycogen storage disease	Single gene	No positive results
F	1.1	Metabolic myopathy	Mitochondrial disease	Single gene	No positive results
M	0.7	Metabolic myopathy	Mitochondrial disease	Single gene	No positive results
M	6	Metabolic myopathy	Mitochondrial disease		
F	11.5	Metabolic myopathy	Mitochondrial disease	WES	*TMEM70*
M	9.7	Metabolic myopathy	Mitochondrial disease	Gene panel	*EARS*
F	4.8	Metabolic myopathy	Glycogen storage disease	Single gene	No positive results
M	0.3	Metabolic myopathy	Mitochondrial disease		
M	3	Metabolic myopathy	Lipid storage disease		
F	7.6	Metabolic myopathy	Mitochondrial disease	MtDNA	No positive results
M	8.5	Metabolic myopathy	Mitochondrial disease	MtDNA	MtDNA T8993C
F	6.5	Metabolic myopathy	Mitochondrial disease	Gene panel	No positive results
F	1.1	Metabolic myopathy	Mitochondrial disease		
M	10.3	Metabolic myopathy	Mitochondrial disease	Gene panel	*TSFM*
M	1.3	Metabolic myopathy	Mitochondrial disease	MtDNA	No positive results
M	4.9	Metabolic myopathy	Mitochondrial disease	Gene panel	*NARS2*
M	0.7	Metabolic myopathy	Mitochondrial disease		
M	0.6	Metabolic myopathy	Mitochondrial disease	MtDNA	No positive results
M	12.8	Metabolic myopathy	Glycogen storage disease	Single gene	*GAA*
F	4.6	Metabolic myopathy	Mitochondrial disease	Gene panel	*KARS*
M	8	Metabolic myopathy	Mitochondrial disease	Single gene	*COL4A1*
F	1.6	Metabolic myopathy	Glycogen storage disease		
M	10.4	Metabolic myopathy	Mitochondrial disease		
F	5.5	Neurogenic changes			
M	11.7	Neurogenic changes		Single gene	No positive results
F	4.9	Neurogenic changes			
M	3.3	Neurogenic changes		Single gene	*SMN1*
M	1	Neurogenic changes			
F	10.4	Neurogenic changes		Single gene	*SMN1*
M	5.2	Neurogenic changes		Single gene	No positive results
M	6.7	Neurogenic changes			
M	11.9	Neurogenic changes			
M	3.4	Neurogenic changes			
F	11.5	Neurogenic changes			
F	9.6	Neurogenic changes		Single gene	*SMARD1*
F	3.2	Neurogenic changes			
F	7.4	Neurogenic changes		Single gene	*SMN1*
M	16.5	Neurogenic changes		Gene panel	*TRPV4*
M	10.8	Neurogenic changes		Single gene	No positive results
F	15.1	Neurogenic changes		WES	No positive results
M	0.6	Neurogenic changes		Single gene	No positive results
F	10.3	Neurogenic changes		Gene panel	*TRPV4*
M	9.6	Neurogenic changes		WES	*PRUNE*
F	7.2	Neurogenic changes		WES	*TBCD*
F	3.5	Neurogenic changes		Single gene	*SMN1*

*A-DG, alpha-dystroglycan; WES, whole exome sequencing*.

### Prevalence Analysis in the Different Age Groups

We further subclassified results on the basis of the age at which the biopsy was performed, as follows:

55 biopsies from infants from 0 to 3 months.
37 biopsies from infants between 3 and 6 months.
105 biopsies from infants between 6 and 18 months.

Interpretation of muscle biopsy in infants below age 3 months is very challenging due to the small diameter of fibers, whereas that of biopsy from older children has greater chance to be specific.

Among the 55 samples from infants of 0–3 months, 29 had a biopsy with non-specific alterations or nearly normal (52.7%); three had a biopsy consistent with muscular dystrophy (5.4%); five showed alteration consistent with a congenital myopathy (9%); 16 reported a condition of a metabolic or mitochondrial myopathy (29.1%); and two were consistent with neurogenic muscle damage (3.6%).

Among the 37 sampling performed on infants of 3–6 months, 14 had a biopsy with non-specific alterations or nearly normal (37.8%); four had a biopsy consistent with muscular dystrophy (10.8%); seven showed alteration consistent with a congenital myopathy (18.9%); five reported a condition of a metabolic or mitochondrial myopathy (13.5%); and seven were consistent with neurogenic muscle damage (18.9%).

Among the 105 sampling performed on infants of 6–18 months, 49 had a biopsy with non-specific alterations or nearly normal (46.7%); 16 had a biopsy consistent with muscular dystrophy (15.2%); seven showed alteration consistent with a congenital myopathy (6.7%); 20 reported a condition of a metabolic or mitochondrial myopathy (19%); and 13 were consistent with neurogenic muscle damage (12.4%).

### Results of Ancillary Procedures

Immunofluorescence (IF) analysis of sarcolemmal proteins was performed in 53 muscle biopsies. In six cases, a defect of merosin was detected; in seven cases, a defect of alpha-dystroglycan was detected; two cases had a defect of collagen 6; one case had dystrophin; and one case had sarcoglycans.

A biochemical assay of the mitochondrial complexes activity was performed on 31 biopsy samples.

In 21 cases, we detected alteration of the respiratory chain enzymes including reduction in the activity of complex IV, increased activity of citrate synthase, or reduced activity of complex I.

Seven of these patients subsequently obtained a genetic diagnosis.

In three muscle biopsies showing features of a congenital myopathy without pathognomonic elements, ultrastructural investigation with electron microscopy was performed. In two cases, we observed the presence of nemaline bodies that were not clearly evident at standard histopathology stainings, thus allowing a diagnosis of a nemaline rod myopathy.

### Genetic Outcome

In our cohort, in a total of 94 patients, we performed at least one genetic investigation [43 single-gene test, 13 mtDNA sequencing, 24 next-generation sequencing (NGS) with genes panel, and 14 NGS with whole exome sequencing (WES)].

Among patients with specific histopathological results, on 70, we performed at least one genetic analysis according to the indication from the biopsy findings. Conversely, on only 24 patients with unspecific or normal results did we perform genetic analysis.

Interesting, on 14 patients, we performed NGS (seven with indicative histopathology and seven with unspecific results) and in particular WES analysis with seven conclusive results.

In the whole, 42 genetic diagnoses of neuromuscular disorders (NMDs) were achieved including nine mitochondrial diseases. Further four genetic diagnoses were actually not related to a neuromuscular condition.

**Figure 2:**
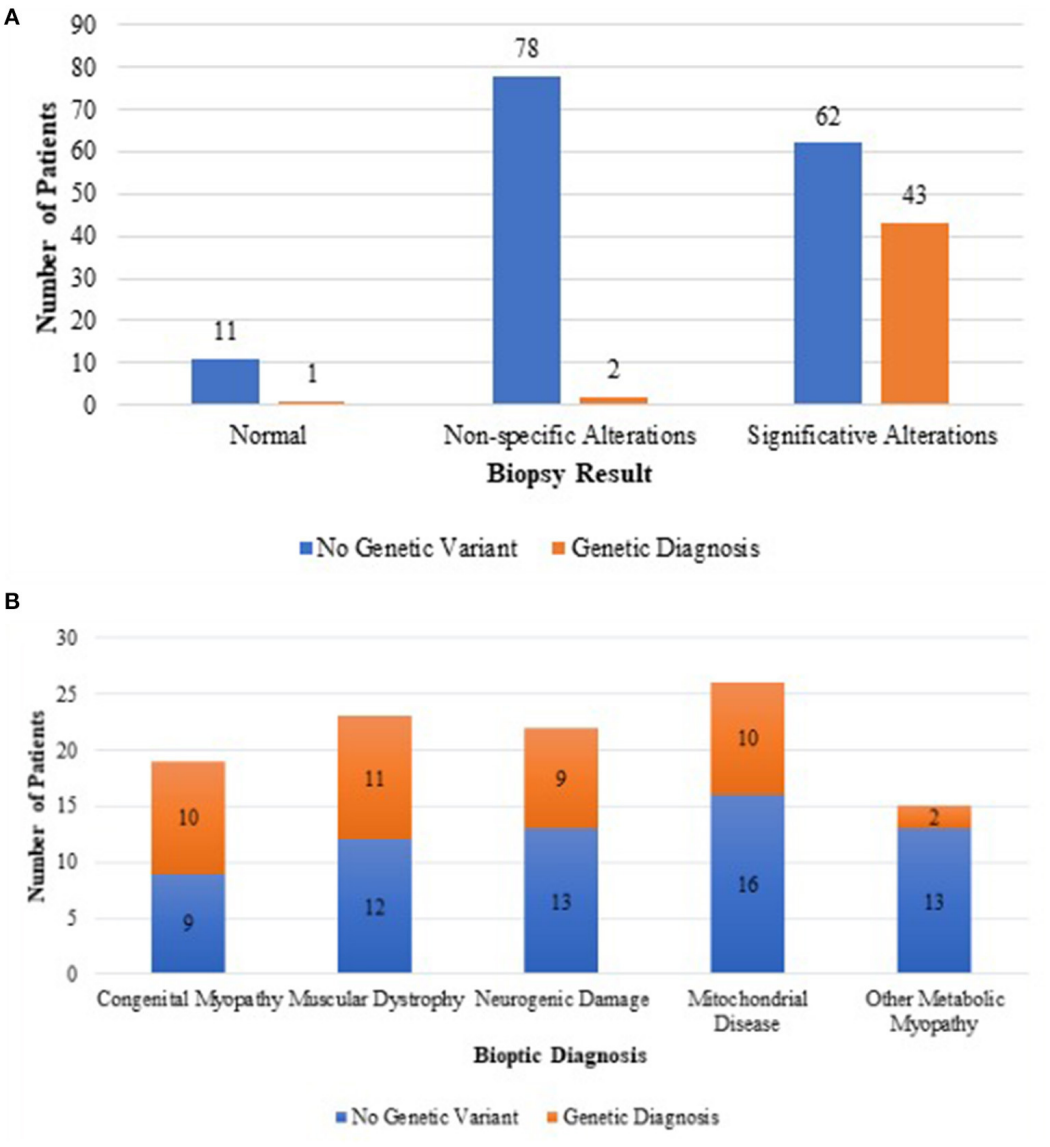
Difference in genetic outcome between unspecific muscle biopsies and biopsies suggesting a definite histopathological diagnosis **(A)**. Genetic outcome of each category of biopsy suggesting a definite histopathological diagnosis **(B)**.

Forty (90%) genetic results were consistent with the histopathology findings and only three from non-specific or normal biopsies (Figure 2).

In the group of congenital muscular dystrophies, 11 patients received a genetic diagnosis with mutations in the following genes: *LAMA2* (three cases); *POMGNT1* (one case); *COL6* (one case); *TTN* (one case); *GMPPB* (one case); *POMT2* (one case); *POMGNT2* (one case); *DMD* (one case); and *SCGA* (one case). In all cases, the Immunofluorescence (IF) study results were consistent with the underlying genetic defect except for the case carrying mutation in titin gene (*TTN*).

Among the patients whose biopsy was indicative of a mitochondrial myopathy, we reached seven genetic diagnoses including one case with mutation in mtDNA (T8993C), *AGK* (two cases), *TSEN54* (one case), *EARS* (one case), *TSFM* (one case), and *NARS2* (one case).

Mutations in POLG (one case), *TSEN54* (one case), *TMEM70* (one case), and *KARS* (one case) were eventually identified only after the biochemical data, as the histopathology study did not clearly indicate a mitochondrial myopathy.

In the group of metabolic myopathies related to glycogen accumulation, the following genetic diagnosis were made: *PYGM* (one case) and *GAA* (one case).

Among patients whose biopsy was indicative a congenital myopathy, 10 received a genetic diagnosis with identification of pathogenic mutations in the following genes: *MTM1* (three cases), *RYR1* (one case), *ACTA1* (one case), *TNNT1* (one case), *TPM3* (one case), *TTN* (one case), *CFL2* (one case), and *DNM* (one case).

In the group of neurogenic damage, nine patients received a genetic diagnosis carrying pathogenic mutations in the following genes: *SMN1* (four cases), *IGHMBP2* (one case), *TRPV4* (two cases), *PRUNE* (one case), and *TBCD* (one case).

Among patients with unspecific alterations or nearly normal biopsy, three had a genetic nature of different origin: one congenital central hypoventilation syndrome (CCHS) with mutation of *PHOX2B* gene, one neurodegenerative disorder Rett-like syndrome, with mutation in *CDKL5*, and one a *CCP1* gene mutation.

Considering the different age groups, among the 55 sampling performed on infants of 0–3 months of age, six genetic diagnoses were reached (10.9% of the total); among the 37 patients who underwent muscle biopsy between 3 and 6 months of age, 10 received genetic diagnosis (27%); and in the group of 105 patients who underwent a muscle biopsy between 6 and 18 months of age, 29 received a genetic diagnosis (28.6%).

## Discussion

In this retrospective study, we evaluated the diagnostic value of muscle biopsies in infants presenting with hypotonia in the first year of life and its possible role in guiding further investigations based on the histological findings. We also measure the consistency of muscle biopsy findings with the subsequent genetic findings. The study has several limits: first, detailed clinical information is not available, as we inferred only the clinical suspect and the final biopsy report. In addition, not all the biopsies subsequently underwent the ancillary procedures (immunofluorescence studies, biochemical analysis, and electron microscopy). Lastly, given its retrospective nature and the limited availability of follow-ups, genetic results are not standardized. Thus, we are unable to provide statistical significance to positive genetic result. However, we want to stress that cases with specific or indicative muscle biopsy results are more likely to undergo genetic tests, and in most cases, the results from the two investigations are consistent.

Several studies have analyzed the role of muscle biopsy in the diagnostic process of neuromuscular pathologies in pediatric age (12–15). However, only a few studies have focused on the role of biopsy in infant hypotonia diagnostic flow chart (14). The evaluation of muscle biopsy in such an early age can be more challenging, as it is expected to have peculiar histopathological characteristics, such as a physiological increase in the adipose connective tissue and greater centralization of the nuclei.

Yang et al. (12) reviewed muscle biopsies performed in pediatric population with suspected NMDs reported that muscle biopsy identified abnormal pathologic findings of significance in 62.9% of cases, and the pathologic result alone led to a clinical diagnosis in 33.9%. The most common histopathology results were type 1 predominance, denervation, and type 2 atrophy; however, the children performing muscle biopsy in the Yang study (12) often presented with multiple signs and symptoms of NMD involvement.

Gibreel et al. (13) showed specific pathologic diagnoses in 60% of cases, which helped make a clinical diagnosis in 33% of cases. Histological results were completely normal in 45 patients (27%). Minimal abnormalities not sufficient to make a definitive pathologic diagnosis were reported in 23 patients (14%). Also, in this study, presenting symptoms were multiple, including seizures, abnormal gait, and elevated CK, and the median age population was 7 years.

Thavorntanaburt et al. (14) attested that definitive abnormal pathologic findings were present in 80.3% of cases, and muscle biopsy results changed the clinical diagnostic suspect in one-third of patients. Similarly Sujka et al. (15) demonstrated that in 39% of cases, the diagnosis was modified after muscular biopsy.

Our study overall disclosed comparable outcomes to the most recent pediatric literature on the topic. In our cohort of a sample of 197 biopsies, 46.7% showed no significant alterations, while in 53.3% of cases, the biopsy proved to be decisive in addressing a diagnostic suspect. Half of these biopsies eventually led to a genetic diagnosis. Conversely, in two cases only, the result of the histological examination was disproved by the outcome of the genetic examination: a patient with apparently mitochondrial damage in which a mutation in *COL4A1* was subsequently found and one with biopsy indicative of congenital muscular dystrophy with mutation in titin gene (TTN). *COL4A1* mutation leads to a brain angiopathy with susceptibility to hemorrhages (16). The reason for a secondary mitochondrial alteration in our patient remains unexplained. The histopathological spectrum of titin defect present with an extended range of alterations including myofibrillar damage, cores and minicores, internal nuclei, cores, necrosis, and fibrosis (17).

The group in which the muscle biopsy showed the highest degree of agreement with the genetic results was that of congenital myopathies: among 19 histological samples indicative of a congenital myopathy, in 10 cases (53%), the data were confirmed by subsequent genetic testing, showing a high accuracy of the biopsy findings. The ultrastructural investigation with electron microscopy also proved fundamental. In two cases, we observed the presence of nemaline bodies that were not clearly evident at the standard histopathology staining, thus successfully leading the genetic investigations. The first patient had a mutation in *TNNT1* gene that encodes the slow twitch skeletal muscle isoform of troponin T; the second patient had a very rare mutation in *CFL2* gene that encodes Cofilin 2, a member of a group of proteins that regulate actin-filament dynamics. Nemaline bodies can be absent at the early stage of the disease, and sometimes, a second biopsy is required (18).

In the other groups, the concordance rate between biopsy and genetic findings was 42% for congenital muscular dystrophies, 38% for neurogenic myopathies, and 21% for metabolic myopathies.

It must be specified that Duchenne/Becker Muscular Dystrophy and SMA are routinely excluded by straightforward genetic investigations without the need of muscle biopsy; thus, the prevalence of these common disorders cannot be calculated from our study.

Interestingly, we identified a case with generalized hypotonia and markedly elevated CK in which muscle biopsy was performed in the patient with suspected congenital muscular dystrophy. Accordingly, muscle biopsy showed primitive muscle damage, and immunofluorescence analysis revealed a significant reduction in dystrophin, compatible with dystrophinopathy, which was confirmed by molecular analysis.

Muscle histology of SMA in the very first months of life of can be confusing; thus, SMN1 screening is routinely performed in all patients of our cohort before muscle biopsy. Interestingly, in four patients with SMN1 deletion, muscle biopsy was performed before the genetic outcome. All these patients presented histopathology signs of neurogenic changes.

Most interestingly, in a patient with two normal copies of *SMN1* gene, muscle biopsy findings of neurogenic damage triggered further genetic investigation with Sanger sequencing of *SMN1* gene and allowed to identify homozygous point mutation in *SMN1* gene, which is a unique result with profound implications for the current therapeutic opportunity.

Excluding the abovementioned case, neurogenic findings are not uncommon among our cohort, and it is remarkable that several very rare and novel diagnoses were made from biopsies specifically showing a neurogenic damage including *PRUNE, TRPV4*, and *TBCD* gene mutations. This indicates the importance of muscle histology in leading the genetic investigation of a non-5q-related form of infant motor neuron disease.

The most common histopathological diagnosis in the whole cohort was that of metabolic pathology and in particular a mitochondrial defect.

Secondary metabolic alterations are a common finding in muscle biopsy of the neonatal period. Lipid and glycogen accumulation for instance can be related to parenteral nutrition in the severe cases or selective tube feeding (8).

In our cohort, metabolic alterations are particularly frequent in muscle biopsy taken from infants in the very first months of life (group 0–3 months) if compared with incidence in the other age groups, and it is noteworthy that the rate of genetic diagnosis in youngest infants is scarce.

Lastly, among the biopsies initially classified as non-specific, additional methods such as biochemical analysis or electron microscopy made it possible to reach at a genetic diagnosis. In these cases, the clinician should target further investigations, which must be always considered in the diagnostic workup of muscle biopsy approach.

Normal biopsies are still to be taken into account in ruling out a primitive muscle condition and directing the clinicians toward differential diagnosis. In two patients with normal muscle histology, a definitive genetic diagnosis of Ondine syndrome and Rett-like syndrome led by the clinical picture confirmed the non-muscular origin of the disease. A further patient with non-specific myopathology at routine staining was diagnosed with a congenital myasthenic syndrome based on electrophysiological tests and a positive response to acetylcholinesterase inhibitors.

## Conclusion

The multidisciplinary workup in the diagnosis of a floppy infant with a suspect NMDs is to piece together information from clinical, electrophysiological, and histochemical tests to target the molecular genetic investigations, as guided by rational diagnostic algorithms that are largely missing for these conditions.

It has been found that muscle biopsy can lead to a diagnosis, help the clinician in the choice of a genetic test, or even modify a diagnosis made previously (14).

## Data Availability Statement

The raw data supporting the conclusions of this article will be made available by the authors, without undue reservation.

## Ethics Statement

Ethical review and approval was not required for the study on human participants in accordance with the local legislation and institutional requirements. Written informed consent to participate in this study was provided by the participants' legal guardian/next of kin.

## Author Contributions

MV and CF: acquisition of data, interpretation of data, drafting the manuscript, revision of the manuscript, and final approval to the final version to be published. PB, SB, MT, AD, SS, RF, MM, MPe, CP, GP, MPa, AM, LR, LN, CM, and CB: acquisition of data, interpretation of data, revision of the manuscript, and final approval to the final version to be published. All authors contributed to the article and approved the submitted version.

## Funding

This work has been partially supported by AFM grant 22431. This work has been supported by Ministero Salute Ricerca Corrente 2021.

## Conflict of Interest

The authors declare that the research was conducted in the absence of any commercial or financial relationships that could be construed as a potential conflict of interest.

## Publisher's Note

All claims expressed in this article are solely those of the authors and do not necessarily represent those of their affiliated organizations, or those of the publisher, the editors and the reviewers. Any product that may be evaluated in this article, or claim that may be made by its manufacturer, is not guaranteed or endorsed by the publisher.
